# Mortality and ventilatory effects of central serotonin deficiency during postnatal development depend on age but not sex

**DOI:** 10.14814/phy2.14946

**Published:** 2021-07-06

**Authors:** Gary C. Mouradian, Madeline Kilby, Santiago Alvarez, Kara Kaplan, Matthew R. Hodges

**Affiliations:** ^1^ Department of Physiology Medical College of Wisconsin Milwaukee WI USA; ^2^ Neuroscience Research Center Medical College of Wisconsin Milwaukee WI USA

**Keywords:** 5‐HT, respiratory development, serotonin, tryptophan hydroxylase 2

## Abstract

Serotonin (5‐HT) influences brain development and has predominantly excitatory neuromodulatory effects on the neural respiratory control circuitry. Infants that succumb to sudden infant death syndrome (SIDS) have reduced brainstem 5‐HT levels and Tryptophan hydroxylase 2 (Tph2). Furthermore, there are age‐ and sex‐dependent risk factors associated with SIDS. Here we utilized our established Dark Agouti transgenic rat lacking central serotonin KO to test the hypotheses that CNS 5‐HT deficiency leads to: (1) high mortality in a sex‐independent manner, (2) age‐dependent alterations in other CNS aminergic systems, and (3) age‐dependent impairment of chemoreflexes during post‐natal development. KO rat pups showed high neonatal mortality but not in a sex‐dependent manner and did not show altered hypoxic or hypercapnic ventilatory chemoreflexes. However, KO rat pups had increased apnea‐related metrics during a specific developmental age (P12–16), which were preceded by transient increases in dopaminergic system activity (P7–8). These results support and extend the concept that 5‐HT per se is a critical factor in supporting respiratory control during post‐natal development.

## Graphical abstract

CNS serotonin is important for breathing, especially during development. We expand our understanding of physiologic and biochemical changes in breathing and respiratory brain centers at specific ages caused by a lack of CNS serotonin synthesis in a rat model.

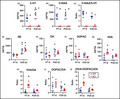

## 1 INTRODUCTION

Serotonin (5‐HT) influences neural circuit maturation during pre‐ and post‐natal development and is an important neuromodulator in multiple CNS circuits in adult mammalian life (Cummings & Hodges, 2019; Daubert & Condron, 2010; Deneris & Gaspar, 2018; Dosumu‐Johnson et al., 2018; Gaspar et al., 2003; Okaty et al., 2015). Neurons that produce 5‐HT arise from distinct neural segments in the developing hindbrain and migrate to midline and ventrolateral aspects of the brainstem, where they produce and release multiple excitatory neuropeptides and 5‐HT to modulate neuronal excitability in their target fields (Brust et al., 2014; Corcoran et al., 2009; Hodges & Richerson, 2010; Okaty et al., 2015). 5‐HT neurons are thought to influence respiratory control through their largely excitatory tonic modulation of the respiratory network, and through putative intrinsic cellular CO_2_/pH sensitivity as central respiratory chemoreceptors (Brust et al., 2014; Cerpa et al., 2017; Iceman et al., 2013). However, the role of CNS 5‐HT in respiratory control during post‐natal development remains unclear.

Sudden infant death syndrome (SIDS) is the leading cause of post‐neonatal mortality in human infants (1 to 12 months old). Rare prospective data from infants that later succumbed to SIDS indicate a predisposition for ventilatory dysfunction, including obstructive and central apneas, decreased hypoxic and hypercapnic ventilatory chemoreflexes (Kahn et al., 1992; Kato et al., 2001) and impaired arousal from sleep (Kato et al., 2003). Further, the average arterial PCO_2_ and PO_2_ levels causing sleep arousal are significantly higher and lower, respectively, in near‐miss SIDS cases compared to control infants (Hunt, 1981). SIDS occurs most frequently from 2 to 4 months of age (Hakeem et al., 2015), more frequently in male versus female infants, and most frequently during sleep (Hakeem et al., 2015). Important advances in our understanding of potential pathophysiological mechanisms include careful examination and comparisons of brainstem tissues from SIDS and control infants, which led to the identification of reduced brainstem 5‐HT tissue levels and reduced brainstem tryptophan hydroxylase 2 (rate‐limiting enzyme in 5‐HT synthesis) levels among other findings (Bright et al., 2017; Duncan, 2010; Kinney, 2009; Kinney et al., 1983, 2001, 2003, 2005). Thus, SIDS may result from a combination of central 5‐HT deficiency and ventilatory dysfunction during specific developmental ages, during which males may be at highest risk.

Rodent models of CNS 5‐HT deficiency (due to germline mutations preventing or limiting 5‐HT neuron formation, global 5‐HT production, or chemical lesions of 5‐HT neurons) indicate that the brainstem 5‐HT system is critical for respiratory control, especially during development (Cummings et al., 2011a; Davis et al., 2019; Dosumu‐Johnson et al., 2018; Erickson et al., 2007; Hodges et al., 2009; Kaplan et al., 2016; Young et al., 2017). 5‐HT deficient mouse models that lack most or all 5‐HT neurons (and thus 5‐HT) show higher perinatal mortality rates. Pet‐1 null and conditional Lmx1b null mice, which lack most or all 5‐HT neurons, respectively, also have reduced resting (eupneic) breathing, chemoreflexes, breathing instability, and apneas, and impaired autoresuscitation and hypercapnia‐induced arousal from sleep (Cummings, Hewitt, et al., 2011; Dosumu‐Johnson et al., 2018; Hodges et al., 2009). While informative, the limitation of these studies is that 5‐HT neurons, and all the neurotransmitters and neuropeptides they produce and release, fail to develop. Indeed, Tph2‐/‐ mouse models have contributed to our understanding of 5‐HT *per se* on respiratory function, but these studies lack chemoreflex assessment, are limited to a few ages, and/or lack an evaluation of histologic or neurochemical changes within the brainstem (Alenina et al., 2009; Chen et al., 2013; Cummings, Commons, et al., 2011). Thus, while these studies point to a major role for 5‐HT neurons to support vital homeostatic ventilatory control mechanisms during development, the role(s) of 5‐HT on the control of breathing during development remain incompletely understood.

Rats have well‐defined developmental brainstem neuroanatomy, particularly within several key nuclei with known contributions to the control of breathing. A series of published semi‐quantitative immunohistochemical datasets from a single lab have shown that several anatomic, neurochemical, and functional shifts naturally occur within the brainstem at or between postnatal day 12 and 14 (P12–14), which may predispose the respiratory control system to potential failure and may thus represent a critical window of development (Gao et al., 2011; Liu and Wong‐Riley, 2002; Liu & Wong‐Riley, 2010a; Liu and Wong‐Riley, 2008,; Liu & Wong‐Riley, 2010b). Based on our current understanding of this developmental window of vulnerability in rats, and the need to establish the role of CNS 5‐HT in respiratory control during post‐natal development, we developed a rat model of selective CNS 5‐HT deficiency *via* a zinc‐finger nuclease‐mediated mutation in the Tph2 gene of Dark Agouti rats (DA*^Tph2^* ^−/−^ rats; referred to as KO rats hereafter)(Kaplan et al., 2016). These rats selectively lack CNS 5‐HT as adults but retain the neurons that would otherwise have been serotonergic ((Tph2‐/‐; Ddc+/+) raphe neurons). Initial characterization of these KO rats showed that they have high mortality early during postnatal life and around the second week of life at a time where they have frequent apneas during active sleep, but normal ventilatory chemoreflexes (Kaplan et al., 2016; Young et al., 2017). Remarkably, these rats also showed eupneic ventilatory dysfunction only during specific developmental ages (P0–2 and P12–15), but also showed periods of respiratory “stability” in the intervening ages (Kaplan et al., 2016). The mechanisms leading to the periodic normalization of breathing and predisposition of breathing instability at P12–15 remain unknown in this model. Furthermore, it is unknown if male KO rats are more susceptible to post‐natal mortality relative to females (as seen in human SIDS), or if ventilatory chemoreflexes at specific developmental timepoints are altered. To address these questions and further elucidate the developmental role of CNS 5‐HT in respiratory control, we studied young KO rats to test the hypotheses that CNS 5‐HT deficiency: (1) causes mortality in a sex‐dependent manner, (2) leads to age‐dependent changes in other aminergic systems, and (3) have age‐dependent impairment hypoxic and hypercapnic breathing responses.

## 2 MATERIALS AND METHODS

### 2.1 Animals

All rats were housed in the Biomedical Resource Center at the Medical College of Wisconsin, were maintained on a 12:12‐h light–dark cycle, and given food and water *ad libitum*. KO rats were generated by Het x Het breeding (DA^Tph2+/−^ x DA^Tph2+/−^), from which 94 pups were used in this study. Data collected were used for all analyses even if the animal succumbed to death mid‐way through the study. DA rats heterozygous for the Tph2 deletion were used for breeder pairs and showed normal parental behaviors and pups displayed normal feeding behaviors (milk spots in the abdomen) as previously noted (Kaplan et al., 2016). Heterozygote rats had no physiologic or neurochemical differences compared to wild‐type (WT) rats (27) and were thus grouped together. All experiments and protocols were approved by the MCW Institutional Animal Care and Use Committee.

### 2.2 Ventilatory measurements and analyses

Ventilation in neonatal rats was measured using a custom‐built, 200mL Plexiglass plethysmograph as described previously (Kaplan et al., 2016; Mouradian et al., ,^2012^, ^2019^). Briefly, the gas inflow rate (150 ml/min) was balanced at the same or slightly lower than outflow vacuum rates to prevent CO_2_ accumulation and assure rapid gas exchange. The chamber temperature was maintained at 29–30°C for P1–9 animals and 27°C for P10–22 with a heated aluminum floor (Dyna‐sense; Scientific Instruments). Chamber temperature (27–30°C; Warner Instruments), relative humidity (HX15; Omega), and pressure (Validyne differential pressure transducer) were measured throughout data acquisition. Analog signals were converted through a 16 channel A/D converter and digitally sampled at 200 Hz and recorded through WinDaq data acquisition software. Breathing was recorded, while rats were exposed to 20 min of room air (21% O_2_, balanced N_2_), followed by either 10 min of acute hypoxia (12% O_2_, balanced N_2_) or 10 min of acute hypercapnia (21% O_2_, 7% CO_2_, balanced N_2_). The last half of each condition was used for data analyses. Animal temperatures were measured after each experiment using a T‐type rectal thermocouple probe and reader (Omega).

All data collected were analyzed offline using LabChart Pro Software as described previously (Mouradian et al., 2019). In brief, each study was recorded in its own file, opened in LabChart, and two additional channels were added to measure the voltage deflection from peak to valley of each respiratory cycle (tidal volume) and the rate of respiratory cycles (breathing frequency). Tidal volume was measured in volts and converted to a volume using a volume calibration. Tidal volume (V_T_) was then corrected for animal and chamber temperature, relative humidity, and atmospheric pressure (Drorbaugh & Fenn, 1955; Hodges et al., 2002), and is expressed as ml/breath/100g of body weight. Analyzed data were devoid of apneas, sighs, sniffing, and movement artifacts. Minute ventilation (V_E_; ml/min/100g) was calculated as breathing frequency (f; breaths/min) times V_T_. No sex differences (main effects of sex: *p* > 0.05) were measured for either breathing frequency or V_T_ in a sampling (n = 4–7 per sex per genotype) of WT and KO rats at each age range studied (main effects: age *p* < 0.05,) and therefore data across sexes are pooled.

Ventilatory data from the last half of the 20 min of room air condition and the last 5 min of the 10‐min challenge conditions were manually analyzed for spontaneous apnea‐like events defined by the cessation of breathing greater than the average of one respiratory cycle to identify possible breathing pattern abnormalities. These apnea‐like events or respiratory pauses were analyzed by an investigator blinded to animal genotype. The duration of each apnea‐like event was from the end of the first breath to the start of the following breath. The total number, average length, and the sum duration of time of apneic‐like events were calculated for each animal and the average of each metric per animal per group is reported.

### 2.3 High‐performance liquid chromatography with electrochemical detection

WT and KO rat pups aged P7–8 (n = 7, 6, respectively) or P20–22 (n = 6, 7, respectively) were anesthetized with isoflurane, decapitated, and the brain tissues were rapidly removed. The brainstem was separated from the rest of the tissue and frozen (−80C) for high‐performance liquid chromatography (HPLC) measurements of norepinephrine (NE), dopamine (DA), dihydrophenylacetic acid (DOPAC), serotonin (5‐HT), 5‐hydroxyindoleacetic acid (5‐HIAA), and homovanillic acid (HVA) as described previously (Kaplan et al., 2016).

### 2.4 Statistics

Statistical analyses were performed using GraphPad Prism (version 8). The normality of data distributions was verified using the D’Agostino‐Pearson normality test. A Chi‐square Gehan–Breslow–Wilcoxon test was used to compare survival curves. Two‐tailed *t* tests and two‐way ANOVAs were used to determine the main effects and interactions with Sidak's post hoc tests when appropriate. Significance thresholds were *p* < 0.05. Data are expressed as mean ± standard deviation.

## 3 RESULTS

### 3.1 Mortality by genotype and sex

**FIGURE 1:**
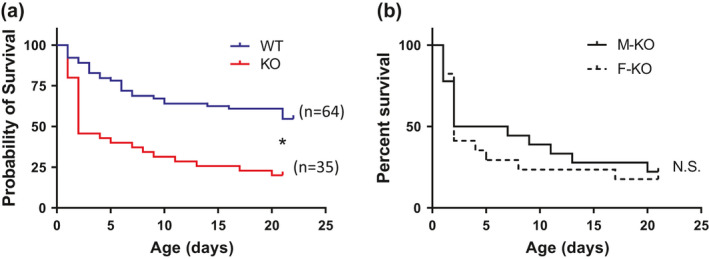
KO rats have increased mortality without any sex differences. (a) Survival (% of pups born per genotype) was reduced to ~50% by postnatal (P) day 2 in KO rats which steadily declined with wild‐type survival up to P21. (b) There were no sex differences in survival rates of KO rats. *p < 0.05 by chi‐square test. WT, n = 64; HM, n = 35 (18 F and 17 M)

The survival rate at P2 was ~50% for KO rats (vs. ~80% for WT) and steadily declined to ~20% by P21 (vs. ~55% for WT; Figure 1a). Stratification of the survival rates by sex was then performed since males have a greater risk for SIDS than females (Hakeem et al., 2015). Male KO rats had a P2 survival rate of ~50% (vs. ~89% for male WT) and a P21 survival rate of ~22% (vs. ~67% for male WT). Female KO rats had a P2 survival rate of ~41% (vs. ~73% for female WT) and a P21 survival rate of ~18% (vs. ~38% for female WT). Male versus female KO rat survival rates were similar across development (Figure 1b). Thus, sex does not appear to be a determining factor for mortality rates in KO rats.

### 3.2 *Hypoxic and hypercapnic ventilation and body temperature is reduced in KO rats*.

**FIGURE 2:**
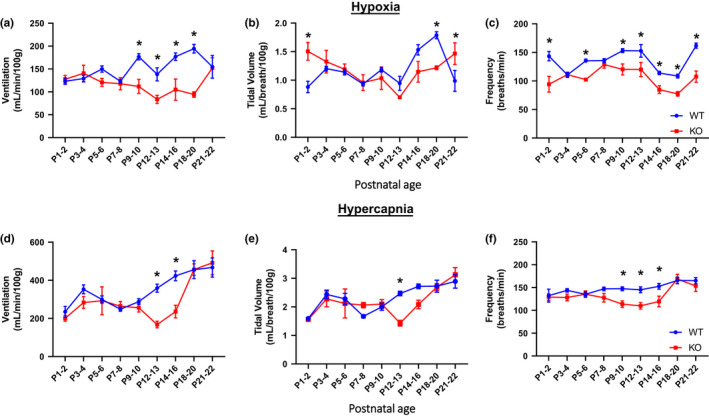
Hypoxic and hypercapnic ventilation are reduced in KO rats within specific developmental age ranges. Minute ventilation (ml/min/100g; a), tidal volume (mL/breath/100g; b) and breathing frequency (breaths/min; c) during hypoxic breathing conditions (12% O_2_) are lower in KO rats (n = 7–11) versus WT rats (n = 8–22) from postnatal days 9–20 driven almost exclusively by lower breathing frequencies (except from P18 to P20 where tidal volume is also lower). Minute ventilation (d), tidal volume (e), and breathing frequency (breaths/min; f) during hypercapnic breathing conditions (7% CO_2_) are lower in KO rats (n = 5–16) versus WT rats (n = 8–24) from postnatal days 12–16 driven by lower tidal volumes from P12 to P16 and lower breathing frequencies from P14 to P16. Note that hypoxic and hypercapnic ventilation are all lower from P12 to P 16. *p < 0.05, two‐way ANOVA with Sidak post hoc test

We had previously shown that eupneic (room air) ventilation was reduced in KO rats at specific post‐natal ages. Adding to this, data set with more studies yielded a similar data set with KO rats having lower eupneic breathing from P9 to P20 (Table 1). Total ventilation in KO rats during acute hypoxia was also reduced compared to WT rats (main effects: genotype and age *p* < 0.05) from P9 to P20 mainly due to a reduced breathing frequency (main effects: age and genotype *p* < 0.05), and there were lower tidal volumes from P18 to P20 (Figure 2a‐c; main effects: age and interaction *p* < 0.05). Body temperature in KO rats during acute hypoxia was also reduced at P3–4 and P12–22 (Figure S1a; main effects: interaction, age, and genotype *p* < 0.05), despite warming the chamber floor (29–30°C; P1–9, and 27°C; P10–22) in an attempt to normalize any thermoregulatory deficiency (Kaplan et al., 2016). Ventilation during acute hypercapnia was also lower in KO rats compared to WT rats (main effects: age *p* < 0.05) from P12 to P16 due to lower tidal volumes (main effects: age *p* < 0.05) from P12 to P13, but lower breathing frequencies (main effects: age and genotype *p* < 0.05) from P9 to P16 (Figure 2d‐f). Body temperature in KO rats during acute hypercapnia was also reduced from P14 to P16 (Figure S1b; main effects: interaction, age, and genotype *p* < 0.05). However, when the hypoxic ventilatory response (HVR) or acute hypercapnic ventilatory response (HCVR) was expressed as a percentage to the baseline breathing of the same animal (percent of control), fewer differences among KO rats and controls were found across age. The HVR and HCVR increased with age similar to previous reports in rats (Davis et al., 2006), and was similar at each age for both KO and WT rats (Table 1) with a few exceptions where the HVR was lower at P21–22 in KO rats and the HCVR was increased in KO rats. The sum of the data indicated that ventilatory chemoreflexes were relatively unaffected across development, consistent with previous findings at 2 weeks of age (Young et al., 2017) or as adults (Kaplan et al., 2016), but lower breathing during hypoxia and hypercapnia are at least in part likely driven from reductions in body temperature in KO rats.

**TABLE 1:** Comparison of average room air (RA) minute ventilation (second column; as ml/min/100g), hypoxic ventilatory response (HVR; third column; expressed as a percent of breathing during the challenge relative to room air breathing × 100%), and hypercapnic ventilatory responses (HCVR; fourth column; percent of breathing during the challenge relative to room air breathing × 100%) for WT versus KO rat pups across age ranges studied (indicated on the left column)

Age	RA		HVR		HCVR	
	WT	KO	WT	KO	WT	KO
P1–2	126.8 ± 6.4	108.3 ± 13.1	98.2 ± 26.2	81.4 ± 23.9	141.9 ± 26.3	180.3 ± 44.2
P3–4	196.4 ± 10.4	150.2 ± 13.1	76.3 ± 8.8	88.1 ± 19.4	171.5 ± 17.6	205.5 ± 16.9
P5–6	166.4 ±6.9	157.9 ± 17.8	92.4 ± 11.9	80.4 ± 6.5	174.2 ± 14.8	198.5 ± 30.5
P7–8	122.3 ± 4.6	122.2 ± 7.8	135.7 ± 20.8	109.3 ± 7.9	190.2 ± 18.9	196.8 ± 33.8
P9–10	181.6 ± 6.7*	118.6 ± 12.3	86.3 ± 10.2	112.6 ± 18.0	178.7 ± 16.1	188.0 ± 22.4
P12–13	159.2 ± 15.7*	54.6 ± 4.8	125.3 ± 52.3	143.2 ± 44.6	195.0 ± 25.1	265.8 ± 65.5
P14–16	165.5 ± 4.8*	103.9 ± 17.3	114.2 ± 21.0	130.4 ± 43.6	247.3 ± 68.7	288.6 ± 79.2
P18–20	146.2 ± 5.0*	78.7 ± 6.2	140.1 ± 23.3	127.2 ± 51.1	300.6 ± 97.5	549.3 ± 282.0
P21–22	119.7 ± 12.9	130.1 ± 11.9	205.1 ± 53.4*	136.3 ± 31.1	311.2 ± 95.6*	344.4 ± 86.6

* *p* < 0.05 for WT versus KO within the corresponding age range.

#### 3.2.1 Brainstem amines and metabolites during development

**FIGURE 3:**
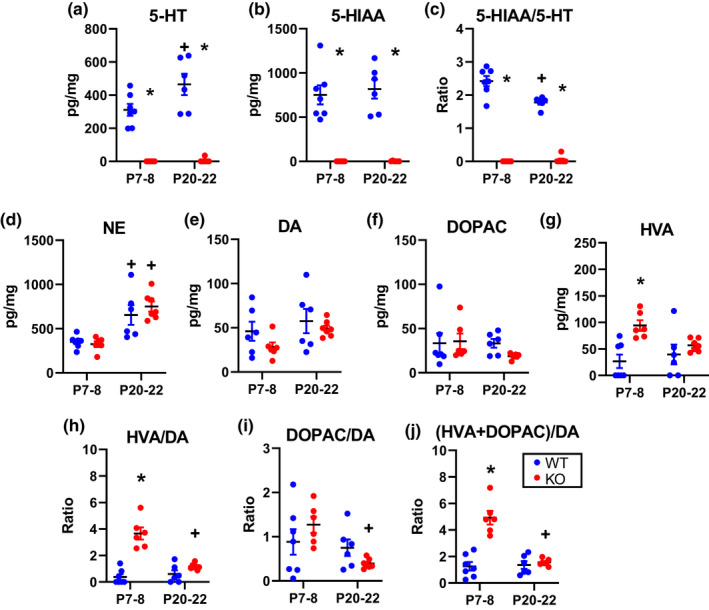
Neurochemicals within the brainstem tissue of KO rats are altered during development. KO rats lack serotonin (5‐HT; a), its metabolite (5‐HIAA; b), and a 5‐HT turnover ratio (c; 5‐HIAA/5‐HT) in brainstem tissue rats aged P7–8 and P20–22. Norepinephrine (NE; d), dopamine (DA; e), and its metabolite, DOPAC (f) are similar between genotypes within P7–8 and P20–22 ages. A metabolite of DA and NE, Homovanillic acid (HVA; g), is significantly greater in KO rats versus WT rats from P7 to P8 but not from P20 to P22. The increase in HVA increased the dopamine turnover ratio (HVA/DA; h). DOPAC/DA ratio is unchanged between genotypes (i). The dopamine turnover ratio inclusive of both dopamine metabolites (HVA +DOPAC/DA) is elevated from P7 to P8 but not P20 to P22 in KO rats (j). *p < 0.05 between genotype within age; +*p* < 0.05 across age within genotype, two‐way ANOVA

Our prior studies confirmed that adult KO rats lack brainstem 5‐HT and HIAA (5‐HT metabolite), while otherwise retaining “serotonergic” (DDC‐expressing) neurons without altering dopamine (DA) or norepinephrine (NE) (Kaplan et al., 2016). Here we tested if the loss of central 5‐HT caused compensatory changes to other amines during development *via* HPLC to measure brainstem tissue levels of 5‐HT, NE, DA, and metabolites 5‐HIAA, DOPAC, and HVA. Consistent with our prior report, KO rats lacked brainstem 5‐HT (main effect: genotype *p* < 0.05) or 5‐HIAA (main effect: genotype *p* < 0.05) in tissue homogenates collected at P7–8 and P20–22 (Figure 3a‐c). The levels of NE (main effect: age *p* < 0.05) and DA (no significant main effects) were similar between KO and WT rats at each age and NE but not DA increased with age (Figure 3d‐e). There were no differences in DOPAC (no significant main effects), the dopamine‐specific metabolite (Figure 3f;). However, P7–8 KO rats had a significantly greater level of HVA (no significant main effects) a major catecholamine metabolite for DA, which increased the HVA/DA turnover ratio (main effects: genotype and interaction *p* < 0.05)(Figure 3g‐h; no significant main effects). The dopamine DOPAC/DA turnover ratio significantly decreased with age in KO rats unlike that in WT rats (Figure 3i; no significant main effects). When accounting for both dopamine metabolites, the dopamine turnover ratio was significantly elevated in P7–8 KO rats which were reduced to WT levels by P20–22 (Figure 3j; main effects: age, genotype, and interaction *p* < 0.05). Together, these data show that Tph2 knockout has some effects on other aminergic systems which may indicate potential compensatory changes in dopaminergic signaling in response to depleted central 5‐HT early in post‐natal development.

#### 3.2.2 *Apnea*‐*like events during control and hypoxic conditions in developing KO rats*

**FIGURE 4:**
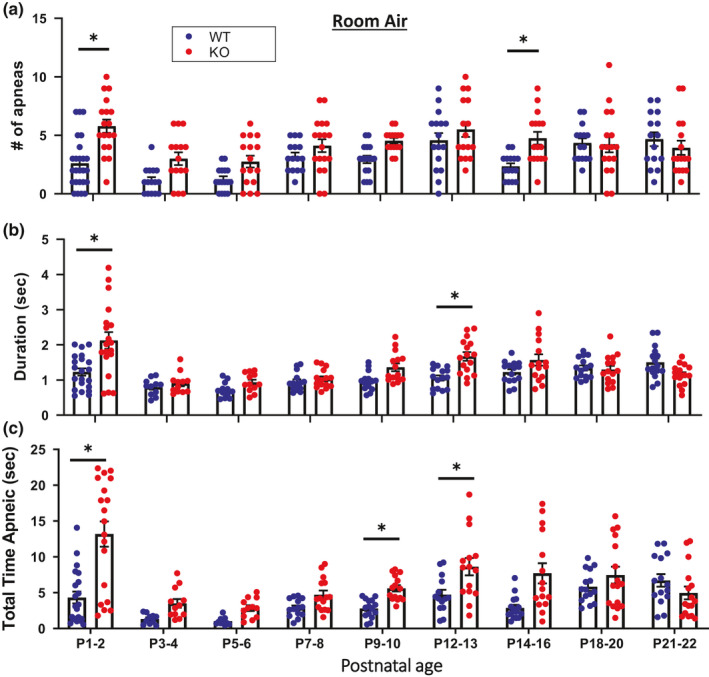
Apneic‐like metrics measured during room air breathing were greater during two distinct developmental ages in KO rats. The number of apneic‐like events (#; a), average duration (b), and the total duration of apneic‐like events (c) were greater from P1 to P2 and from P12 to P13 or P14 to P16 in KO (n = 14–18) versus WT (n = 15–24) rats. Note, the last 10 minutes from room air ventilation studies were used for analysis. *p < 0.05 between genotype within age, two‐way ANOVA with Sidak post hoc test

**FIGURE 5:**
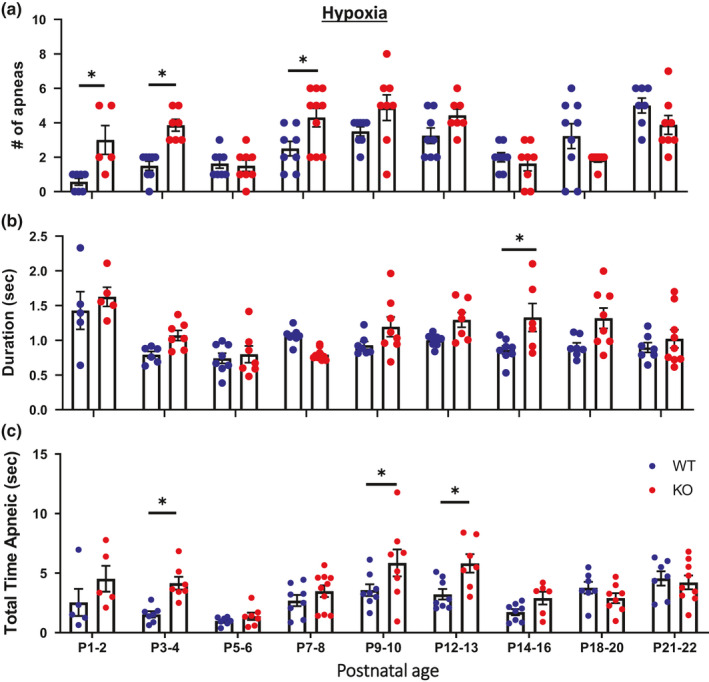
Apneic‐like metrics measured during acute hypoxia were greater during two distinct developmental ages in KO rats. The number of apneic‐like events (#) was greater from P1 to P2 in KO (n = 5–10) versus WT (n = 7–9) rats (a) whereas the average duration (b) and the total duration of apneic‐like events (c) were greater from P12 to P13 or P14 to P16 in KO versus WT rats. Note, the last 5 minutes of acute hypoxic ventilation studies were used for analysis. * *p* < 0.05 between genotype within age, two‐way ANOVA with Sidak's post hoc test

Our prior study indicated that KO rats have disrupted breathing rhythms during eupneic ventilation based on increased variability measures (Poincare analyses) from P1 to P2 and P12 to P14 (Kaplan et al., 2016). Here we further tested whether the number of respiratory pauses or apneas (as defined in the Materials and Methods), average duration of apneas, and the total time spent apneic under room air, hypoxic, and hypercapnic conditions was altered during development in KO rats. Under room air conditions, KO rats had more apneas from P1 to P2 and P14 to P16 (Figure 4a; main effects: age and genotype *p* < 0.05), longer average apnea durations from P1 to P2 and P12 to P13 (Figure 4b; main effects: age, genotype, and interaction *p* < 0.05), and a greater total time spent apneic from P1 to P2 and P9 to P13 compared to WT rats (Figure 4c; main effects: age, genotype, and interaction *p* < 0.05). During acute hypoxic challenges, KO rats compared to WT rats had more apneas from P1 to P4 and P7 to P8 (Figure 5a; main effects: age and interaction *p* < 0.05), longer average apnea durations from P14 to P16 (Figure 5b; main effects: age *p* < 0.05), and greater total time spent apneic from P3 to P4 and P9 to P13 (Figure 5c; main effects: age and genotype *p* < 0.05). There were no differences nor any statistical main effects in the number of apneas, average apnea duration, or total time spent apneic between KO and WT rats during hypercapnic challenges. Thus, CNS serotonin deficiency led to age‐dependent apnea and disruption of eupneic ventilation during development which persists during hypoxic conditions.

## 4 DISCUSSION

The brainstem serotonin (5‐HT) system is embedded within and provides critical excitatory neuromodulatory influences within the respiratory control network, in addition to central CO_2_/pH chemoreception and temperature regulation(Hodges & Richerson, 2008a). Infants that succumb to SIDS have reduced brainstem 5‐HT levels that likely results from a host of 5‐HT system abnormalities (Bright et al., 2017; Duncan, 2010; Kinney et al., 1983). Whereas many animal studies have investigated the impact of 5‐HT neuron deficiency on respiratory development, the impact of 5‐HT deficiency *per se* on breathing, chemoreflexes, and mortality throughout post‐natal development, and whether sex is a differentiating factor, remain unclear. Here, we tested the impact of CNS 5‐HT deficiency on the control of breathing in a genetically engineered rat that lacks Tph2 (KO) (Kaplan et al., 2016). Our data indicate that complete CNS 5‐HT deficiency leads to: (1) high neonatal mortality in a sex‐independent manner, (2) no changes in the hypoxic or hypercapnic ventilatory responses, but decreased body temperature, ventilation, and apnea‐like events at rest and during respiratory challenges in an age‐dependent manner, and (3) transient (potentially compensatory) increases in brainstem dopaminergic system activity.

Several lines of evidence support the overall concept that the brainstem 5‐HT system plays at least two major roles in the control of breathing. First, 5‐HT neurons project to several nodes within the respiratory control network and provide a tonic, excitatory, neuromodulatory drive to breathe (Hodges & Richerson, 2008a). Second, 5‐HT neurons play a major role in facilitating the hypercapnic ventilatory chemoreflex through an intrinsic pH/CO_2_ sensitivity (in a subset of 5‐HT neurons) and by tonic excitation of other important sites for central chemoreception such as the retrotrapezoid nucleus (RTN) (Yuanming et al., 2019). Our data are consistent with the concept that 5‐HT neurons support eupneic breathing during development but point to specific ages in which their contributions may be greatest. KO rats showed an increased incidence of apnea‐like events during room air breathing and during exposure to hypoxia within the first 1–4 days after birth, consistent with reductions in eupneic breathing during this age reported in our previous study (Kaplan et al., 2016). It is also during this period in early postnatal development in which mortality rates were highest in both male and female KO rats (~70–88% of total mortality occurs in the first post‐natal week), suggesting that CNS 5‐HT deficiency provides a vital input to cardiorespiratory systems early in neonatal life. This is supported by others reporting that genetically or pharmacologically induced 5‐HT loss in mice causes severe impairments to the cardiorespiratory system in an age‐dependent manner (Chen et al., 2013; Yang and Cummings, 2013). In our KO model, the greatest increase in mortality occurs from P1 to P4, an age when apnea‐like ventilatory disruptions were greatest and minute ventilation was lowest. Taken with the observation that later in development when breathing during hypoxia and hypercapnia are most reduced in KO versus WT rats, the data are consistent with the concept that 5‐HT is critical for maintaining eupneic breathing very early in neonatal life (P1–4) and modulates chemoreflexes later in postnatal development.

KO rats also showed decreases in breathing during hypoxia and hypercapnia and increased values for apnea‐like measures at various ages between P9 and P20, which may or may not reflect changes to metabolic rate given the body temperature in KO rats is reduced at all but P9–10 and P12–13 in acute hypoxia and hypercapnia, respectively. The most consistent age range demonstrating such changes was after the second week of life, which corresponds with our prior report of reduced eupneic breathing observed between P12 and P16 (Kaplan et al., 2016). Thus, the reduction in ventilation after the first postnatal week may indicate a generalizable reduction in respiratory network excitation, even during environmental challenges, pointing to a critical role of 5‐HT in maintaining excitatory respiratory network activity and/or reflect a reduction in metabolism given that CNS 5‐HT is important in thermoregulation. Furthermore, the putative “critical window” of normal respiratory control system development has been isolated to ~P12–13 in rats (Gao et al., 2011; Liu et al., 2009; Liu & Wong‐Riley, 2010a; Wong‐Riley et al., 2019) and is characterized by transient declines in excitatory signaling and a concomitant increase in inhibitory signaling throughout the respiratory network (Wong‐Riley et al., 2019). Therefore, 5‐HT deficiency combined with the natural shifts in excitation and inhibition may have a particularly detrimental effect and may unmask a critical role for 5‐HT *per se* during the second postnatal week, and provides additional mechanistic insights into the pathophysiology of human SIDS which also appears to be highly age‐dependent. Alternatively, the 5‐HT may not have any additive impact on the putative critical window of development given that differences in ventilatory measures between WT and KO rats are observed well before P12–13. Future experiments are needed to dissect the potential interplay between 5‐HT deficiency and ventilatory changes during the putative critical window of respiratory development, and interactions between metabolic rate, breathing, and CNS 5‐HT.

Despite the deficiencies in eupneic ventilation (Kaplan et al., 2016), CNS 5‐HT deficiency *per se* did not appear to influence ventilatory chemoreflexes across postnatal development. Previous measures of the ventilatory responses to mild hypoxia (F_I_O_2_ = 0.17) and hypercapnia (F_I_CO_2_ = 0.05) in KO rats at 2 weeks of age indicated no effect of CNS 5‐HT deficiency (Young et al., 2017), similar to adult KO rats (Kaplan et al., 2016). Here, we showed that the hypoxic and hypercapnic ventilatory responses (expressed as a percentage of control breathing) in KO rats were equivalent to WT rats throughout the development. The lack of effect of CNS 5‐HT deficiency on chemoreflexes during development contrasts previous studies using genetic deletion of 5‐HT neurons, where Lmx1b^f/f/p^ mice show a blunted CO_2_ chemoreflex at P21 and as adults, and reduced hypoxic chemoreflexes at P12, P21, but normal responses as adults (Cerpa et al., 2017; Hodges & Richerson, 2008b; Hodges et al., 2008). This discrepancy points to 5‐HT neurons, but not necessarily 5‐HT *per se*, as having larger contributions to the CO_2_ and hypoxic chemoreflex during the development. However, it is noteworthy that metabolic rate measures were not made during the current studies, where differences in metabolic rate responses to these challenges may have influenced the results. Taken together, these findings suggest that 5‐HT *per se* is a major source of the 5‐HT system's contribution to the excitatory facilitation of resting breathing during postnatal development and may contribute minimally to ventilatory chemoreflexes during the first ~2.5 weeks of life in rats, consistent with the concept that medullary 5‐HT neurons “switch from tonic respiratory drive to chemoreception” after the completion of postnatal development (Cerpa et al., 2017).

The effects of 5‐HT deficiency on eupneic breathing were age‐dependent, where there were periods of “normalization” of resting ventilation after P4 until P9, and after P20 into adulthood (Kaplan et al., 2016). The respiratory control network undergoes major developmental shifts in neurochemicals and receptors throughout development in rats (Gao et al., 2011; Liu & Wong‐Riley, 2010a, 2010b; Wong‐Riley et al., 2019), including other aminergic systems, which may compensate for the constitutive loss of 5‐HT during these age ranges in KO rats. Indeed, HPLC analysis of brainstem homogenates from P7 to P8 KO rats indicates a significant and transient upregulation of dopaminergic signaling in KO rats relative to WT rats. Dopaminergic signaling extends throughout the bulbar respiratory network and has largely excitatory effects on breathing (Lalley, 2008). For example, genetically induced loss of dopaminergic neurons in mice severely disrupts breathing and hypoxic responsiveness leading to death within 24 hours of birth (Nsegbe et al., 2004). Application of apomorphine, a non‐selective dopamine receptor agonist with specificity to mainly D2‐like receptors, increases ventilation and the HCVR whereas blockade of CNS dopamine receptors reduces phrenic nerve discharge (Lundberg et al., 1979, 1982; Nielsen & Bisgard, 1983). Thus, increases in dopamine turnover could indicate an increase in excitatory dopaminergic signaling as a mechanism to compensate for the loss of 5‐HT excitatory neuromodulation between the P5 and P8 age range or simply be a compensatory response to keep DA levels in KO rats at WT levels. Whether this increase in dopaminergic signaling is a compensatory mechanism present at earlier or later ages, or if there are additional compensatory factors remains to be tested.

In conclusion, our data support and extend the concept of 5‐HT *per se* as a critical factor supporting vital respiratory control mechanisms during postnatal life. Constitutive 5‐HT deficiency in KO rats led to deficits in resting breathing and body temperature at specific ages without affecting chemoreflexes and further led to high postnatal mortality rates which did not differ among males and females. Thus, 5‐HT deficiency *per se* may not represent the only biologic abnormality underlying SIDS pathophysiology but may contribute to a multi‐factorial but 5‐HT system‐related syndrome resulting from more than one abnormality. 5‐HT has a critical excitatory neuromodulatory role for respiratory control during key periods of postnatal development and is likely a contributing factor to a multifactorial biological dysfunction in human SIDS.

## CONFLICTS OF INTEREST

All authors claim no conflicts of interest.

## AUTHOR CONTRIBUTIONS

GCM: Designed the study, completed studies, analyzed the data, and prepared and edited the manuscript. SAA: completed experiments, analyzed the data, edited the manuscript. KK: Completed studies and analyzed the data. MK: Completed studies, analyzed the data, and edited the manuscript. MRH: Designed study, prepared and edited the manuscript.

## Supporting information



Fig S1
