# Supplementary figures and images for: Mortality and ventilatory effects of central serotonin deficiency during postnatal development depend on age but not sex
Source: Physiol Rep. 2021 Jul 6;9(13):e14946. doi: 10.14814/phy2.14946 (PMC8259800; doi:10.14814/phy2.14946)

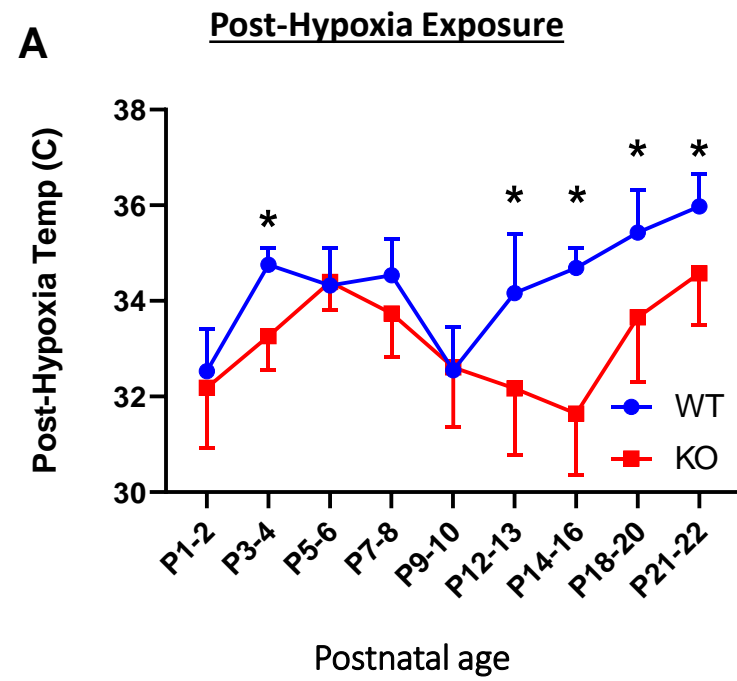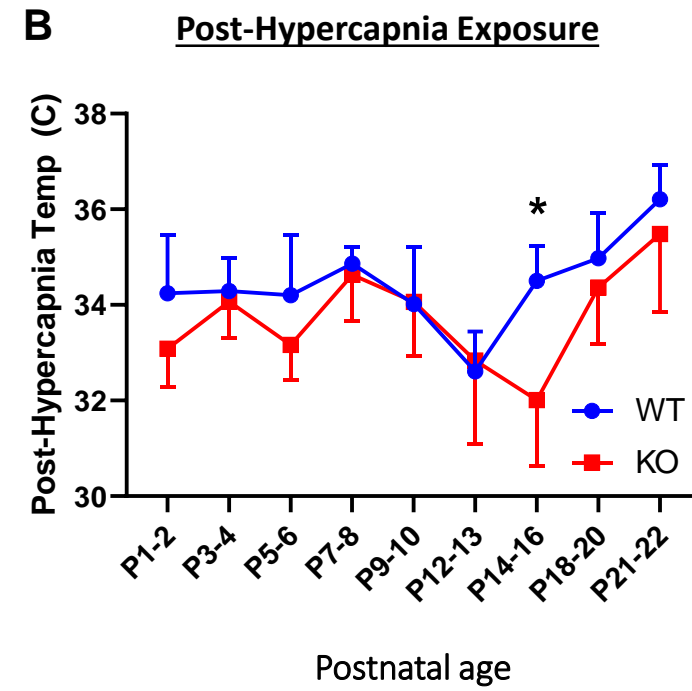

Supplement: Supplementary file 1 — Fig S1 [file PHY2-9-e14946-s001.pdf]
